# Comparison of Mast Cell Density and Prognostic Factors in Invasive Breast Carcinoma: A Single-Centre Study in Malaysia

**DOI:** 10.21315/mjms2023.30.5.7

**Published:** 2023-10-30

**Authors:** Norashikin Awang Ahmad, Shau Kong Lai, Roslina Suboh, Huzlinda Hussin

**Affiliations:** 1 Department of Pathology, Faculty of Medicine and Health Sciences, Universiti Putra Malaysia, Selangor, Malaysia; 2 Department of Pathology, Hospital Tuanku Ja’afar, Negeri Sembilan, Malaysia; 3 Department of Pathology, Hospital Sultanah Nur Zahirah, Terengganu, Malaysia; 4 Lablink Medical Laboratory, Kuala Lumpur, Malaysia

**Keywords:** mast cells, CD117, breast carcinoma, oestrogen receptor, prognostic factors

## Abstract

**Background:**

Mast cells influence tumour growth, neo-angiogenesis and the propensity for metastasis by contributing to innate and adaptive immune responses in the tumour microenvironment. The number of mast cells has increased in various malignant tumours and their abundance has been associated with either a favourable or unfavourable prognosis. This study investigated the significant difference in stromal mast cell density among multiple prognostic factor groups in invasive breast carcinoma.

**Methods:**

CD117 (c-KIT) antibodies were used to stain 160 formalin-fixed and paraffin-embedded invasive breast carcinoma tissues to demonstrate the presence of mast cells. Then the labelled mast cells were counted in 10 fields at 400× magnification and the mean value was used to represent the mast cell density.

**Results:**

The demographic distribution revealed that most patients were 40 years old or older (92.5%) and of Malay ethnicity (66.3%). With regard to prognostic factors, the most prevalent subtype was invasive carcinoma of no special type (80.6%), followed by tumour grade 3 (41.3%), T2 tumour size (63.1%), N0 lymph node stage (51.3%), presence of lymphovascular invasion (59.4%), positive oestrogen (64.4%) and progesterone receptors (53.1%), and negative human epidermal growth factor receptor 2 (HER2) expression (75.0%). However, there was no significant difference in stromal mast cell density among the different demographic and prognostic factor groups in invasive breast carcinoma.

**Conclusion:**

The findings from this study suggest that stromal mast cells do not play a significant role in preventing or promoting tumour growth in invasive breast carcinoma.

## Introduction

Invasive breast carcinoma is the most commonly diagnosed cancer and the leading cause of cancer death among women. About 2.1 million (11.6 %) newly diagnosed breast cancer cases were reported in 2018, accounting for nearly one in four cancer cases among women globally (1). Breast cancer accounts for 32.1% of all cancer cases among women in Malaysia (2).

Prognostic factors are essential for managing the patients to determine the disease outcome, appropriate treatment modalities and clinical trial design (3). Numerous studies have been conducted in previous years to assess the prognosis of patients diagnosed with invasive breast carcinoma. These studies have examined various clinical and pathological parameters, including patient age, tumour size, tumour type, tumour grade, disease stage, margin status, lymphovascular status, and the status of hormonal receptors (oestrogen and progesterone receptors) and human epidermal growth factor receptor 2 (HER2). Following these studies, several recommendations have been proposed for using various prognostic and predictive factors (4) to assess the prognosis of patients with invasive breast carcinoma.

A close interaction between the tumour and the stromal cells in the tumour microenvironment is necessary for tumour development. Focal alterations in the stroma may create a conducive microenvironment for tumour development. In the tumour microenvironment, mast cells support innate and adaptive immune responses. Mast cells are the first to invade the tumour microenvironment. They release regulatory elements that can subsequently impact tumourigenesis (5).

Mast cells have a dual role, either preventing or promoting tumour growth. Cytokines and proteolytic enzyme secretion by the mast cells induce apoptosis of the neoplastic cells. Mast cells suppress the growth of tumour cells in the fibrotic region of breast cancer by directly killing tumour cells through the action of tumour necrosing factor and indirectly through heparin. The activity of mast cells against tumours is regulated by interleukin-6, −8 and −10 (IL-6, IL-8 and IL-10) and chemokine ligands 3 and 5 (CCL3 and CCL5). It has been suggested that mast cell activation by immunoglobulin E is crucial for anti-tumour immunity. Additionally, mast cells stimulate natural killer (NK), dendritic and T cells to increase anti-tumour activity (6).

In contrast, mast cells may promote tumour proliferation by facilitating tumour angiogenesis. This is achieved through the secretion of heparin-like molecules and various growth factors such as platelet-derived growth factor, vascular endothelial growth factor, stem cell factor and nerve growth factor, which are found in the extracellular matrix. These growth factors stimulate fibroblast growth and angiogenesis, facilitating tumour proliferation. Furthermore, mast cells indirectly release IL-10 and tumour growth factor by interacting with myeloid-derived suppressor cells and regulatory T cells, enhancing their immunosuppressive activity. These dual roles of mast cells in inhibiting and promoting the growth of breast carcinoma warrants further investigation (7).

CD117 (c-KIT) is a type III receptor tyrosine kinase that plays a vital role in signal transduction in various cells. c-KIT is phosphorylated upon binding to its ligand, stem cell factor, initiating a cascade of phosphorylation events that stimulate several transcription factors in different cell types. This activation regulates various cellular processes, such as apoptosis, cell differentiation, proliferation, chemotaxis and cell adhesion. c-KIT-dependent cell types include mast cells, specific haematopoietic stem cells, germ cells, melanocytes, and Cajal cells of the digestive system are all c-KIT-positive, including their tumour cells. Skin adnexal, breast epithelium and specific cerebellar neurons are c-KIT-positive normal cells (8). Normal mast cells are an excellent and almost consistent internal control for c-KIT immunohistochemical staining.

Normal mast cells can be detected by various stains including tryptase, chymase, May-Grunwald Giemsa, toluidine blue and Alcian blue, in addition to c-KIT immunohistochemistry stain (9). Strong c-KIT immunohistochemical expression was observed in all cases of mast cell disease and some cases of serous ovarian carcinoma, malignant melanoma, small cell lung carcinoma and adenoid cystic carcinoma. The strong mast cell membrane c-KIT reactivity is useful for identifying normal mast cells and diagnosing mast cell disorders (10). A study that compared mast cell densities using c-KIT and toluidine blue stains confirmed that c-KIT is more accurate in determining mast cell density in oral submucous fibrosis (11).

This study investigated the role of stromal mast cells in the tumour microenvironment by comparing mast cell density with prognostic factors of invasive breast carcinoma. The results may suggest the prognostic significance of the stromal mast cells in invasive breast carcinoma, which could help improve patient management.

## Methods

### Tissue Sampling and Immunohistochemical Study

This cross-sectional study was conducted in the Histopathology Unit, Department of Pathology, Hospital Tuanku Ja’afar, Negeri Sembilan, Malaysia using 160 archived paraffin-embedded tissue blocks. The sample size for this study was determined based on a study by Glajcar et al. (12), which calculated sample sizes based on the study objectives. The OpenEpi calculator was used to determine sample sizes for each objective and the largest size required, 148, was selected.

A purposive sampling method was used to select cases of invasive breast carcinoma from mastectomy specimens at Hospital Tuanku Ja’afar Seremban between 2015 and 2017. Cases with complete demographic and clinicopathological data were retrieved from the histopathological report in the Laboratory Information System (LIS). The demographic and clinicopathological data collected included age (< 40 years old and ≥ 40 years old), ethnicity (Malay and non-Malay), tumour size (T1–T4), lymph node stage (N0–N3), histologic grade (grade 1–grade 3), histologic type (invasive carcinoma of no special type [NST] and others), lymphovascular invasion (present or absent), oestrogen and progesterone receptor (positive or negative) and HER2 expression (positive or negative). Cases not meeting the selection eligibility criteria were excluded, such as biopsy, wide local excision, and lumpectomy specimens, patients with missing paraffin-embedded tissue blocks, unavailable data in LIS, and incomplete histopathological reports. A cut-off age of 40 years old was used based on a local study in 2011 (13). In recent years, there has been an observed increase in the incidence of breast cancer in younger age groups, despite higher rates still being seen after the age of 50 years old based on the National Cancer Registry 2012–2016.

**Figure 1:**
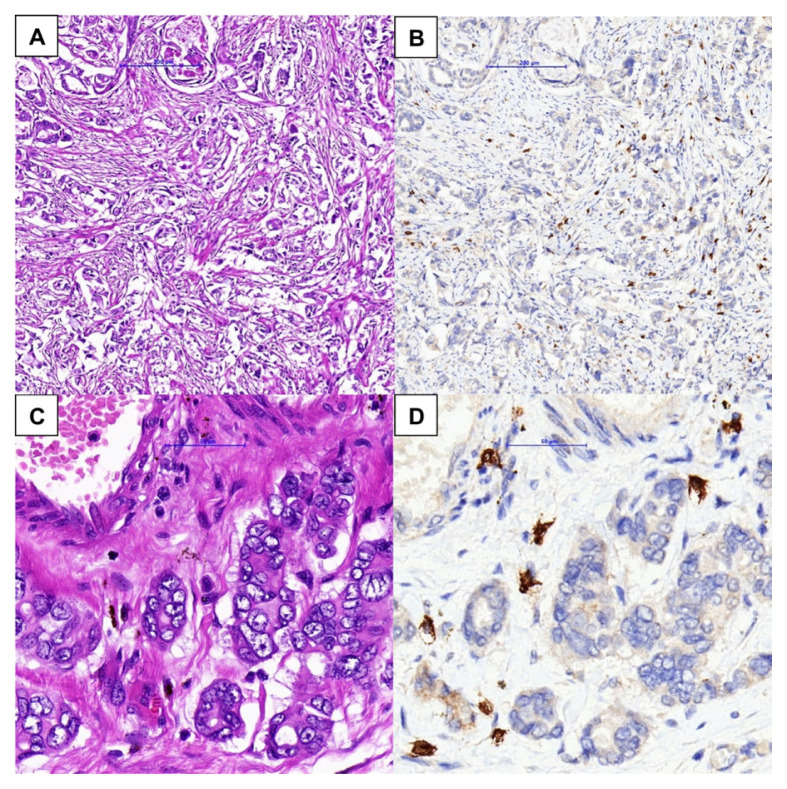
A similar area of invasive breast carcinoma shows mast cells in the background at low magnification, 100× (A: haematoxylin and eosin (H & E), B: c-KIT) and high magnification, 400× (C: H & E, D: c-KIT). The cytoplasm and membrane of the stromal mast cells are highlighted by c-KIT immunohistochemical stain (B and D)

The paraffin-embedded tissue blocks containing tumour cells were cut into sections with a thickness of 3 μm and stained with monoclonal c-KIT antibody (Ventana) using an autostainer (Ventana Benchmark XT) following standard immunohistochemistry staining procedures. The positive control tissue used was the gastrointestinal stromal tumour. The cytoplasm and membrane of mast cells were stained brown at the antigen-antibody binding sites (Figure 1). The slides stained with c-KIT antibody were inspected at low magnification (40*×* and 100*×*) to identify 10 hotspots with high mast cell densities. The mast cells in these 10 hotspots were then counted at high magnification (400*×*) with an ocular grid microscope corresponding to a field of view of 0.55 mm (Olympus BX51; 40*×* objective lens and 10*×* ocular lens; Olympus, Tokyo, Japan). One to three readings were taken from each hotspot, depending on the size of the area. The mast cells were counted randomly in 10 selected areas without apparent hotspots. The density of mast cells was expressed as the mean value based on the formula; a total number of mast cells in 10 hotspot microscopic fields divided by 10. The mast cells were counted simultaneously by a pathologist and a trainee pathologist, who were blinded by the patient’s clinical data. A minimum of 90% agreement was accepted and any percentage discrepancies in slide scoring were immediately reviewed and recounted for consensus.

### Statistical Analysis

Data processing and statistical analysis were conducted using IBM SPSS Statistics for Windows, version 28.0. Mast cell density data were not normally distributed and all variables were presented as the median and interquartile range (IQR) (Table 2). The demographic and clinicopathological data distribution was analysed using frequency analysis (count and percentage) (Table 1). The comparison of mast cell density between groups based on demographic and prognostic factors was analysed by the Mann-Whitney U and Kruskal Wallis tests (Table 2). The *P*-value of < 0.05 was considered significant.

## Results

Most patients (*n* = 148; 92.5%) were 40 years old and above, while the rest (*n* = 12; (7.5%) were under 40 years old. Malay ethnicity was the most frequent, accounting for 66.3% (*n* = 106) of the cases, followed by non-Malay (*n* = 54; 12.5%). Invasive carcinoma of NST (80.6%) and grade 3 (41.3%) were the most prevalent histological types and grades, respectively. T2 tumour size was the largest in percentage (63.1%), followed by T1 (18.1%), T3 (13.8%) and T4 (5.0%). In most cases, lymph node metastasis was not detected (N0) (51.3%). However, lymphovascular invasion was present in 59.4% of patients. Oestrogen and progesterone receptors were positive in more than 50% of the cases, whereas positive HER2 expression only occurred in 25% of the patients (Table 1).

The median stromal mast cell density value was 13.25 (IQR = 12.2) per 1 mm^2^, ranging from 3.1 to 91.6. However, no significant difference was observed in stromal mast cell density among the various demographic and prognostic factor groups associated with invasive breast carcinoma, as shown in Table 2.

## Discussion

Many studies have been done to determine and investigate the role of stromal mast cells in the microenvironment of invasive breast carcinoma. Various research findings suggest that mast cells can serve as a good or a bad prognostic biomarker in breast cancer, which supports the dual roles of mast cells in inhibiting and promoting the growth of breast cancer, as discussed in the introduction.

Studies have shown that the luminal subtype of invasive breast cancer (oestrogen receptor and progesterone receptor-positive) has a significantly higher mast cell density compared to the non-luminal subtype of breast carcinoma (oestrogen receptor and progesterone receptor-negative) (12, 14). Glajcar et al. (12) revealed that mast cell density showed significant positive correlations with oestrogen and progesterone receptors and negative HER2 expression. This study found that mast cell density was correlated with lower tumour grade and cell growth and was negatively associated with tumour size. Findings in this study using mast cells stained with chymase and tryptase suggested the protective role of mast cells in the progression of breast cancer (12).

Another study also demonstrated that high mast cell density in invasive breast carcinoma expressed high levels of hormone receptors. The study postulated that mast cell infiltration is a protective factor in tumour progression caused by mast cell cytolytic activity against malignant cells (15). Oestrogen receptor positivity and intratumoural mast cell density correlated positively (5, 7, 16). Invasive breast cancer with oestrogen receptor positivity has been associated with a favourable prognosis since it suggests a less aggressive tumour. Compared to oestrogen receptor-negative tumours, survival and disease-free duration are relatively long (17). Two extensive studies of 4,444 and 348 cases of invasive breast carcinoma, using c-KIT to stain the mast cells, concluded that stromal mast cells in invasive breast carcinoma were associated with a favourable prognosis (18, 19). Mast cells are considered an independent good prognostic marker and reiterate the role of local inflammatory responses in breast carcinoma development. A study revealed the presence of mast cells in node-negative cases of invasive breast carcinoma. However, there was no significant correlation between mast cell infiltration and histological grade, hormone receptor status or tumour size. Amini et al. (7) concluded that a high number of mast cells in invasive breast carcinoma was associated with low-grade tumours and oestrogen receptor positivity, which are indicators of favourable prognosis in breast carcinoma cases.

On the contrary, a study reported that intratumoural mast cell density was linked to poor prognostic factors in breast cancer, such as positive lymphovascular and perineural invasion (5). The study revealed that stromal mast cells promote tumour vascular invasion and hasten metastatic disease progression. Besides mast cell density, the researchers also examined lymphatic vessel density and found that mast cells play a role in lymphangiogenesis, an essential element of tumour progression. They concluded that intratumoural mast cells have a more significant role in tumour progression than mast cells in other locations. Compared with the present study, cases with positive lymphovascular invasion showed a higher mean rank of stromal mast cell density than negative cases. However, there was no significant difference in stromal mast cell density between groups with lymphovascular invasion or lymph node metastasis.

Another study has shown that an increased number of mast cells in breast carcinoma is associated with a poor outcome. However, the study found no significant association between mast cell number and tumour size, histologic grade, oestrogen receptor expression, progesterone receptor expression and HER2 expression in early breast cancer (20). A statistically significant correlation was found between the negative oestrogen receptor and progesterone receptor with mast cell infiltration (21). During cancer invasion, the primary tumour-microenvironment cell types engaged are cytotoxic T cells, NK cells and mast cells (22). These cells are responsible for the unsatisfactory response to neoadjuvant chemotherapy in breast cancer due to their involvement in the mechanism of treatment resistance (23). The involvement of mast cells in breast cancer angiogenesis at an early stage was also reported (24).

Although we have discussed the positive and negative prognostic implications of stromal mast cells in several studies, this study did not uncover any statistically significant differences in stromal mast cell density between the groups categorised by demographic and prognostic factors of invasive breast carcinoma. Nonetheless, the mean rank of stromal mast cell density was relatively high in several good prognostic factors, namely small tumour size (T1), well-differentiated tumours (grade 1) and positive oestrogen and progesterone tumours (Table 2). None of the similar studies have reported non-significant findings regarding mast cell density and prognostic variables in invasive breast carcinoma. All of these studies were conducted abroad, and the differences in their results could be due to variations in sociodemographic characteristics and biological and genetic behaviours. To the best of our knowledge, this is the first local study investigating the association between stromal mast cell density and prognostic factors in breast cancer. The insignificant study findings in this current study were partly supported by a study of 104 breast cancer cases. Most prognostic factors, such as tumour size, histological grade, TNM stage, progesterone receptor and HER2 expression, showed insignificant differences in mast cell density (5). However, a positive correlation between mast cell density and poor prognostic parameters, such as lymphovascular and perineural invasion and lymph node metastasis, suggests that mast cells participate in both tumour progression and an unfavourable disease course. The differences in their findings with this current study could be attributed to the analysis of both intratumoural and peritumoural areas of mast cell density. Another study revealed an insignificant association between mast cell density and tumour size, lymph node metastasis and HER2 expression in invasive breast carcinoma. However, the overall findings from this study suggest that mast cells played a protective role in inhibiting breast cancer (14). The previous study included some patients who received neoadjuvant chemotherapy, which might have contributed to the difference from the findings of this current study.

Some studies have used tryptase and toluidine blue rather than c-KIT immunohistochemical analysis to detect the presence of mast cells. However, very few studies have compared different types of stains for mast cell detection. It is important to note that in this particular breast cancer tissue, the c-KIT stain on mast cells should be interpreted with caution as it also stains normal breast epithelium. Considering the distinct morphology of both cells, incorrect interpretation was unlikely. However, the haematoxylin and eosin (H & E) stain slides were double-checked in ambiguous areas. These measures were taken to avoid false positive or false negative results.

Our study did not differentiate between the location of stromal mast cells, including intratumoural, peritumoural and non-tumoural areas, which might have influenced the study findings. Previous research has suggested that mast cells in the peritumoural stroma of breast cancer are associated with a favourable prognosis (18, 25). Mast cells were observed in normal breast tissue at varying densities in both the intralobular and interlobular stroma, although they were more noticeable in the interlobular stroma. Earlier studies have indicated that the behaviour of mast cells differs in oestrogen and progesterone-positive and negative invasive ductal carcinoma. Mast cell counts were reportedly greater in the peritumoural region of oestrogen and progesterone-positive invasive ductal carcinoma (16).

## Conclusion

The statistically insignificant difference in stromal mast cell density among various prognostic factor groups of invasive breast carcinoma implies that these cells do not have a clear role in either promoting or preventing tumour growth. Further studies on a larger scale within the same geographic region are necessary to confirm the results of this study.

## Figures and Tables

**Table 1:** Distribution of demographic and prognostic factors in invasive breast carcinoma

Demographic and prognostic factors	*n* = 160	%
Age group (years old)		
< 40	12	7.5
≥ 40	148	92.5
Ethnicity		
Malay	106	66.3
Non-Malay	54	33.7
Tumour size		
T1	29	18.1
T2	101	63.1
T3	22	13.8
T4	8	5.0
Lymph node stage		
N0	82	51.3
N1	30	18.8
N2	29	18.1
N3	19	11.9
Lymphovascular invasion		
Present	95	59.4
Absent	65	40.6
Histologic type		
Invasive carcinoma of NST	129	80.6
Others	31	19.4
Histologic grade		
Grade 1	29	18.1
Grade 2	65	40.6
Grade 3	66	41.3
Oestrogen receptor		
Positive	103	64.4
Negative	57	35.6
Progesterone receptor		
Positive	85	53.1
Negative	75	46.9
HER2 expression		
Positive	40	25.0
Negative	120	75.0

**Table 2:** Comparison of mast cell density between the groups of demographic and prognostic factors

Demographic and prognostic factors groups	*n*	Mast cell density median (IQR)	Mean rank	U^a^	Chi-square (*χ*^2^)^b^	df^b^	*P*-value
Age (years old)							
< 40	12	9.70 (22.3)	69.29	753.50	–	–	0.384^a^
≥ 40	148	13.40 (11.5)	81.41				
Ethnicity							
Malay	106	13.10 (9.5)	77.99	2595.50	–	–	0.336^a^
Non-Malay	54	14.25 (15.7)	85.44				
Lymphovascular invasion							
Present	95	13.30 (12.3)	83.11	2840.00	–	–	0.390^a^
Absent	65	13.20 (13.6)	76.69				
Tumour size							
T1	29	14.10 (12.6)	82.71	–	0.63	3	0.889^b^
T2	101	13.30 (11.5)	81.37				
T3	22	10.35 (18.5)	73.32				
T4	8	11.90 (12.4)	81.31				
Lymph node stage							
N0	82	14.00 (14.5)	80.99	–	0.10	3	0.992^b^
N1	30	12.70 (10.0)	81.70				
N2	29	12.20 (10.0)	78.22				
N3	19	13.70 (8.9)	79.95				
Histologic grade							
Grade 1	29	15.80 (16.8)	85.53	–	1.88	2	0.390^b^
Grade 2	65	12.30 (9.3)	79.88				
Grade 3	66	13.95 (13.1)	79.77				
Histologic type							
Invasive carcinoma of NST	129	13.70 (12.4)	83.29	1640.00	–	–	0.121^a^
Others	31	10.50 (14.1)	68.90				
Oestrogen receptor							
Positive	103	14.10 (15.4)	84.24	2550.00	–	–	0.170^a^
Negative	57	12.30 (9.0)	73.74				
Progesterone receptor							
Positive	85	14.10 (14.1)	82.32	3033.00	–	–	0.597^a^
Negative	75	12.30 (11.2)	78.44				
HER2 expression							
Positive	40	12.15 (11.8)	75.11	2184.50	–	–	0.396^a^
Negative	120	13.85 (12.7)	82.30				

Notes:

a U = Mann-Whitney U test;

b Kruskal-Wallis test
